# Electrocardiogram Interpretation Competency of Primary Health Care Physicians: A Cross-Sectional Study

**DOI:** 10.3390/healthcare13233040

**Published:** 2025-11-25

**Authors:** Abdullah Afif AlShakhs, Abdullah A. AlAmeer, Zahra C. AlEsmaeel, Omniyah AlMotawa, Sajjad M. AlHaddad, Hasan M. AlHaddad, Abdullah Almaqhawi

**Affiliations:** 1 Department of Medicine, College of Medicine, King Faisal University, Al-Ahsa 31982, Saudi Arabia; abdullahafif250@gmail.com (A.A.A.); aa_alameer@hotmail.com (A.A.A.); 2 Department of Medicine, College of Medicine, Imam Abdulrahman Bin Faisal University, Dammam 34212, Saudi Arabia; zahraa.alesmaeel@gmail.com (Z.C.A.); omniyah82002@gmail.com (O.A.); 3 Diabetes Department, King Fahad Medical City, Riyadh 12231, Saudi Arabia; 4 Academy of Family Medicine, Al-Ahsa Health Cluster, Al-Ahsa 36365, Saudi Arabia; dr.hasan.haddad@gmail.com; 5 Department of Family and Community Medicine, College of Medicine, King Faisal University, Al-Hofuf 31982, Saudi Arabia

**Keywords:** electrocardiogram, cardiology, advanced cardiac life support, primary health care physicians, Saudi Arabia, competence, assessment, qualifications, interpretation

## Abstract

**Background:** Effective interpretation of electrocardiogram (ECG) data is a critical skill for primary health care physicians and part of the Family Medicine residency program curriculum. However, there is a lack of data regarding the competency of Primary Health Care Physicians in ECG interpretation in Saudi Arabia. To evaluate the competence of primary healthcare physicians in Saudi Arabia in interpreting common ECG abnormalities. Additionally, it seeks to understand participants’ perspectives on the facilitators and barriers to effective ECG learning. **Methods:** A cross-sectional study was conducted from 28 September 2023 to 1 November 2024. A validated questionnaire was used to assess the knowledge of primary health care physicians. **Results:** A total of 257 physicians participated in the study. Nearly half of the participants (51.8%) attended cardiology rotations and completed the Advanced Cardiac Life Support (ACLS) course. Findings revealed that 74.3% of participants demonstrated poor knowledge of ECG interpretation. Factors significantly contributing to ECG competence included completion of the ACLS course (*p*-value = 0.035), teaching during clinical rotations as a knowledge source (*p*-value = 0.020), and participation in ECG courses (*p*-value = 0.031). Barriers identified encompassed inadequate training programs and inconsistent exposure to ECGs. **Conclusion:** Primary health care physicians demonstrated unsatisfactory performance in ECG interpretation. Completion of ACLS and dedicated ECG courses, as well as exposure to structured teaching during clinical rotations, were significantly associated with higher competency. Therefore, integrating them into core medical curricula is recommended to enhance ECG interpretation skills among primary health care physicians. Further research is warranted to identify the most effective educational strategies for improving competency in this essential clinical skill.

## 1. Introduction

An electrocardiogram (ECG) is a non-invasive test that monitors heart rhythm and electrical activity [1]. It is the most used cardiac investigation due to its simplicity, accessibility, and affordability [2]. Cardiovascular disease (CVD) is the leading cause of mortality worldwide [3]. Early detection through baseline ECG evaluation, particularly in primary healthcare settings, is essential to prevent life-threatening conditions such as cardiac arrest and acute myocardial infarction [4,5]. Primary healthcare physicians serve as the first line of healthcare and play a pivotal role in the diagnosis and management of CVD [6]. A study by Quinn et al. demonstrated that patients who underwent pre-hospital ECG monitoring had significantly lower 30-day mortality rates, attributed to earlier detection of abnormalities [7]. Despite the importance of ECG interpretation skills for healthcare professionals, gaps remain in standard training programs and assessment strategies [1].

Studies in Nigeria have shown poor attitudes and utilization of ECG among Family Medicine residents, while similar research in the United States revealed that ECG interpretation skills of Family Medicine residents do not improve during residency [2,8].

One study in the Qassim region in Saudi Arabia reported ECG competency of primary health care physicians [9]; other local studies involved nurses, interns, and students [10,11,12]. This indicates a gap in knowledge of data on the ECG interpretation competency of primary healthcare physicians in Saudi Arabia, particularly of general practitioners and Family Medicine doctors. Therefore, this study aims to assess the prevalence of ECG interpretation competency among primary healthcare physicians and to identify factors associated with enhanced skill, including exposure to structured training programs and clinical rotations. Strengthening ECG interpretation skills at the primary care level is crucial for improving early diagnosis, enabling timely intervention, and ultimately reducing cardiovascular morbidity and mortality.

## 2. Materials and Methods

### 2.1. Study Design and Population

This cross-sectional study was conducted from 28 September 2023, to 1 November 2024, targeting primary healthcare physicians in the Eastern Province of Saudi Arabia. The study followed the guidelines outlined in the Strengthening the Reporting of Observational Studies in Epidemiology (STROBE) Statement for reporting observational studies (Supplementary Materials) [13].

Inclusion criteria included primary healthcare physicians actively working in the Eastern Province, encompassing both Family physicians at all levels and general practitioners. Physicians who declined to participate or did not complete the questionnaire were excluded. The primary objective was to assess participants’ competence in interpreting common ECG abnormalities. The secondary objective was to identify factors that help effective ECG learning.

### 2.2. Sampling Technique and Data Collection Process

A non-probability convenience sampling technique was employed to recruit participants from various institutions, with the majority affiliated with the Al-Ahsa Health Cluster (39.7%) and the Eastern Health Cluster (28.4%). Data were collected through in-person visits to primary healthcare centers, where researchers personally approached physicians and invited them to participate. Physicians who agreed were provided with access to a structured questionnaire via Google Forms, which they completed on-site. Of the 260 responses obtained, three were excluded due to incomplete answers, resulting in 257 valid responses for final analysis. While this approach enabled accessibility and inclusion from multiple centers, it does limit the generalizability of the findings beyond the sampled population.

### 2.3. Data Collection Tool and Competency Assessment

Data were collected using a structured, pre-validated questionnaire (Coll-Badell et al., 2017 [14]) designed to assess participants’ knowledge of ECG interpretation (Supplementary Materials). The reliability of this instrument was verified via the Intraclass Correlation Coefficient (ICC) in a subsample of 26 nurses, with an ICC above 0.75 indicating excellent reliability. Validity was confirmed by 10 experts (5 physicians and 5 nurses).

The final questionnaire comprised 12 questions: 2 theoretical questions and 10 clinical questions featuring ECG printouts of varying difficulty levels to represent important pathologies. Key content areas included demographics, educational and training background, sources of ECG knowledge, competency assessment, and perceptions of factors facilitating or hindering ECG learning.

For competency assessment, participants were evaluated on their ability to recognize and identify specific ECG abnormalities:

- Q1: Absent P-wave
- Q2: ECG waves and intervals (order and identification)
- ECG1: Atrial flutter
- ECG2: Ventricular fibrillation
- ECG3: Atrial fibrillation
- ECG4: Pathological Q wave
- ECG5: Atrioventricular third-degree block
- ECG6: Bundle branch block
- ECG7: Ventricular tachycardia
- ECG8: Acute myocardial infarction
- ECG9: Normal ECG
- ECG10: Ventricular extrasystole
- ECG11: Atrial tachycardia

All questions were presented in a multiple-choice format, with a single best answer and four options. The 10 clinical ECG questions were based on static images of ECG tracings. After completion, the maximum score (originally 12 points) was converted to a 10-point scale for easier interpretation. A score of 7.5 out of 10 or higher indicated “good” ECG interpretation competency, while scores below 7.5 reflected “poor” knowledge, this threshold was established through agreement among experts (Coll-Badell et al., 2017 [14]).

### 2.4. Data Analysis

Data were analyzed using SPSS software (version 26). Descriptive statistics, including frequencies, percentages, means, and standard deviations, were used to summarize categorical and continuous variables. The chi-square test was applied to examine associations between categorical variables. Multivariable binary logistic regression analysis was performed to identify independent predictors of ECG knowledge. Statistical significance was set at a *p*-value of <0.05.

## 3. Results

A total of 260 accomplished questionnaires were received through google forms. Three of them were excluded due to incomplete answers. Thus, a total of 257 subjects were included in the study.

Table 1 delineates a detailed overview of the participants’ demographic profiles and educational backgrounds. In terms of gender, the participants are almost evenly split, with 54.5% being female (140 individuals) and 45.5% male (117 individuals). Viewing experience years, the majority have less than 5 years at 59.1% (152 individuals), while those with 5–10 years and more than 10 years share similar proportions of 20.2% (52 individuals) and 20.6% (53 individuals), respectively.

The level of the participants was varied, with family medicine residents forming the largest subgroup at 39.3% (101 individuals). General practitioners constitute 30.0% (77 individuals), family medicine consultants 16.7% (43 individuals), and family medicine specialists 14.0% (36 individuals).

The education (program) sector reflects a diversity of institutions from which participants have gained their knowledge. The Al-Ahsa health cluster is the primary source for 39.7% (102 individuals) of the participants’ education. The eastern health cluster is next, representing 28.4% (73 individuals), followed by the Johns Hopkins Aramco healthcare center in Dhahran at 9.3% (24 individuals). Other educational sectors mentioned include King Fahad University hospital in Khobar, health services program in the royal commission in Jubail, and various other sources, which account for percentages ranging from 3.1% to 10.9%.

As for specific training enrollment, 51.8% (133 individuals) of the participants have previously or are currently taking cardiology as part of their training rotations. The completion rate of the advanced cardiac life-support (ACLS) courses is 49.4% (127 individuals), showing a nearly even distribution between those who have and have not completed the course.

The participants reflected a variety of educational methods used to gain knowledge in ECG interpretation knowledge. As 124 participants (48.2%) have attended regular ECG lectures as part of their education, while 133 participants (51.8%) have not. The same number and percentage of participants (124 and 48.2%) reported receiving teaching during clinical rotations. ECG courses are noted as a source of knowledge by 111 participants, which represents 43.2% of the sample; conversely, 146 participants (56.8%) did not report ECG courses as a knowledge source. Self-study using printed materials, such as textbooks, was reported by 99 participants (38.5%), and not reported by 158 participants (61.5%). Finally, self-study using web-based sources was the most reported method, with 134 participants (52.1%) utilizing this resource, while 123 participants (47.9%) did not use online materials for self-study.

**Figure 1:**
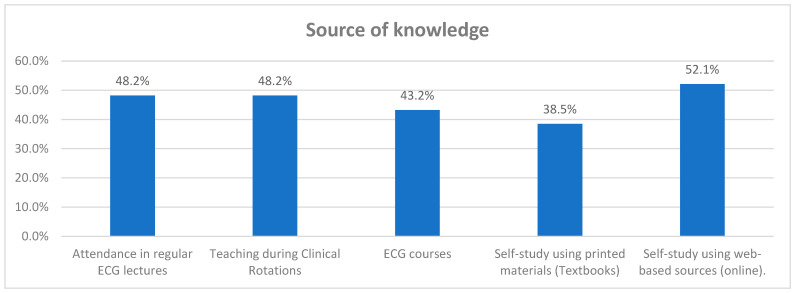
Source of Knowledge for ECG Interpretation.

Figure 1 The participants reflected on a variety of educational methods used to gain knowledge in ECG interpretation. A total of 124 participants (48.2%) reported attending regular ECG lectures as part of their education, while 133 participants (51.8%) did not. The same number and percentage of participants (124; 48.2%) reported receiving teaching during clinical rotations. ECG courses were identified as a source of knowledge by 111 participants (43.2%), whereas 146 participants (56.8%) did not cite ECG courses as a learning source.

Self-study using printed materials, such as textbooks, was reported by 99 participants (38.5%), while 158 participants (61.5%) did not use printed materials for self-study. Finally, self-study using web-based resources was the most frequently reported method, with 134 participants (52.1%) utilizing online materials, compared to 123 participants (47.9%) who did not.

Table 2 categorizes the knowledge level of participants into two classifications based on their overall score from a questionnaire. According to the table, 118 participants are identified as having a good knowledge level, which represents 45.9% of the surveyed population. In contrast, 139 participants fall into the poor knowledge category, comprising a majority of 54.1% of those surveyed. Furthermore, the sample demonstrated level of knowledge falling into mean of 6.67 ± 2.74 SD.

**Figure 2:**
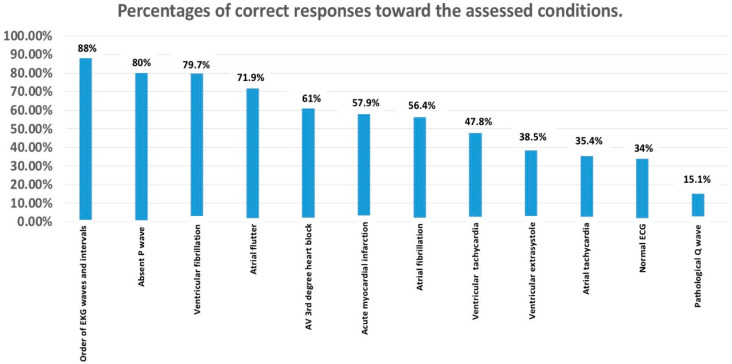
Percentages of correct responses toward the assessed conditions.

Figure 2 illustrates the accuracy of participant responses to a series of electrocardiographic (ECG) conditions. It revealed varied levels of proficiency among participants in identifying specific ECG conditions, with certain areas showing high accuracy and others suggesting the need for improved training and education in ECG interpretation. Waves and ECG intervals received the highest number of correct responses, totaling 227, which suggests a strong understanding of this area among participants. Despite this, there are 30 incorrect responses. For the P wave, a substantial number of correct responses (206) is observed, and (72) incorrect responses.

Atrial fibrillation and Acute myocardial infarction are among the conditions with a higher correct response rate, with 218 and 169 correct answers, respectively. The relatively low number of incorrect responses, 39 for Atrial fibrillation and 88 for Acute myocardial infarction, suggests these conditions are more effectively recognized by the participants.

Conversely, Atrial flutter shows a significant discrepancy, with 112 incorrect responses compared to 145 correct ones. Other conditions such as Ventricular fibrillation, Pathological Q wave, Atrioventricular third-degree block, Bundle branch block, Ventricular tachycardia, and Atrial tachycardia display a mix of correct and incorrect responses, indicating a moderate level of participant knowledge and identification accuracy for these conditions. A notable finding is the higher incidence of incorrect answers (158) for Normal ECG compared to the correct ones (99), which may point to a potential gap in the participants ability to accurately identify normal ECG patterns.

Table 3 provides a clear contrast between what participants perceive as aiding their learning process in ECG interpretation and the obstacles that hinder it. In the Facilitators section, the highest reported factor contributing to ECG learning is Practical case-based training in the program, with 177 participants (68.9%) identifying it as helpful, whereas 80 participants (31.1%) did not find it as such. Extracurricular educational courses are considered facilitators by 127 participants (49.4%), showing an almost even split, with 130 participants (50.6%) not considering them as facilitators. Volunteer work in cardiology clinics is seen as a facilitator by fewer participants, with 70 (27.2%) affirming it versus 187 (72.8%) who do not. A significant portion of participants, 119 (46.3%), believe that Cardiology block important aspects should be adopted and implanted in all blocks, with 138 (53.7%) disagreeing. The Other category for facilitators is the least reported, with only 39 participants (15.2%) marking it, while 218 participants (84.8%) did not.

In the Barriers section, the most reported barrier is the same as the most reported facilitator, Inadequate training in the program, with 177 participants (68.9%) agreeing that it is a barrier, mirroring the number who found practical case-based training to be a facilitator. Dependence on cardiologist expertise is considered a barrier by 86 participants (33.5%), with a majority, 171 participants (66.5%), not considering it a significant barrier. The difficulty of ECG interpretation itself is acknowledged by 120 participants (46.7%) as a barrier, while 137 participants (53.3%) do not report it as such. Lack of resources is reported as a barrier by 78 participants (30.4%), against 179 participants (69.6%) who do not consider it a barrier. The Other category for barriers is the least cited, with only 10 participants (3.9%) reporting it, and a vast majority, 247 participants (96.1%), not identifying with it.

In their description of other, participants in the study identified several key limitations, including inadequate hands-on experience and a deficiency of continuous, quality training. They advocate for ongoing, case-based learning, potentially utilizing digital platforms like WhatsApp for interactive education. Many faces inconsistent exposure to ECGs across different medical roles and specialties, often compounded by the time constraints and heavy workload in clinical settings.

As illustrated in Table 4, significant associations were observed with years of experience, ACLS certification, attendance in regular ECG lectures, teaching during clinical rotations, and participation in ECG courses (*p* < 0.05 for all). Participants with more than 10 years of experience demonstrated the highest proportion of good knowledge (54.7%), compared to those with 5–10 years (28.8%) and less than 5 years of experience (48.7%) (*p* = 0.016). Those who had completed the ACLS course showed a greater proportion of good knowledge (53.5%) than those who had not (38.5%) (*p* = 0.015). Similarly, attendance in regular ECG lectures (53.2% vs. 39.1%; *p* = 0.023), receiving ECG teaching during clinical rotations (52.4% vs. 39.8%; *p* = 0.043), and participation in ECG courses (53.2% vs. 40.4%; *p* = 0.042) were significantly associated with better knowledge levels. No significant associations were found with gender, professional level, cardiology rotation, or self-study habits (*p* > 0.05).

To control for potential confounding effects and identify independent predictors of ECG knowledge, all variables that showed statistical significance in the univariate analysis were entered into a multivariate binary logistic regression model (Table 5). Participants with 5–10 years of experience were significantly less likely to have good ECG knowledge compared to those with more than 10 years of experience (OR = 0.35, 95% CI: 0.15–0.81, *p* = 0.014). Completion of the ACLS course was associated with higher odds of good ECG knowledge (OR = 1.87, 95% CI: 1.09–3.21, *p* = 0.022). Attendance in regular ECG lectures, participation in teaching, and ECG courses showed positive trends but did not reach statistical significance.

Table 5 presents the results of the binary logistic regression analysis examining predictors of good ECG knowledge among participants. After adjusting for demographic and educational factors, three variables remained statistically significant predictors. Participants with 5–10 years of experience were significantly less likely to demonstrate good ECG knowledge compared to those with more than 10 years of experience (OR = 0.24, 95% CI: 0.09–0.64, *p* = 0.004). Conversely, family medicine specialists had significantly higher odds of good ECG knowledge compared to general practitioners (OR = 2.77, 95% CI: 1.05–7.27, *p* = 0.039). Similarly, participants who attended regular ECG lectures showed significantly greater odds of good knowledge (OR = 1.80, 95% CI: 1.02–3.16, *p* = 0.042). Other variables, including gender, professional level (resident or consultant), cardiology rotation, ACLS completion, teaching during clinical rotations, ECG courses, and self-study through printed or online materials, were not significantly associated with ECG knowledge levels.

## 4. Discussion

Electrocardiogram interpretation is an essential skill for all primary health care physicians and forms an integral component of the family physician residency programs [8,15]. Despite its importance, there is a paucity of data on the ECG interpretation competency of primary health care physicians in Saudi Arabia [9]. Therefore, this study aimed to assess ECG interpretation competency among primary healthcare physicians and identify participants’ perceptions of the factors that facilitate or hinder learning.

A two-part questionnaire developed by a research team in Spain was employed, a validated and reliable tool confirmed by subsequent researchers [14]. The study results showed generally poor ECG interpretation competency among primary health care physicians. Atrial fibrillation and acute myocardial infarction were the most recognized conditions, whereas pathological Q waves and normal ECG patterns were the least identified.

Practical, case-based training appeared to be the most significant facilitator for learning ECG interpretation; paradoxically, the most frequently mentioned barrier was inadequate training in these very same hands-on methods. In the univariate analyses, participation in ACLS courses, ECG courses, ECG lectures, and clinical rotation teaching activities was all significantly associated with higher levels of ECG interpretation competency. However, in the multivariable logistic regression model, controlling for confounding factors, participants with 5–10 years of experience were less likely to achieve good ECG competency compared to those with more than 10 years of experience, and being a family medicine specialist and attending regular ECG lectures remained independent predictors of good ECG knowledge. The associations with ACLS, ECG courses, and clinical rotation teaching were not significant.

Our observation of the low competency in ECG interpretation among primary healthcare physicians aligns with most studies on this topic. A study done by Margolis and Reed et al. in a comparison of ECG competency among UAE medical students, Family Medicine residents, general practitioners, and USA Family Medicine residents, reported low levels in both countries [16]. Another study by Mabuza et al. showed strikingly low competency among general practitioners in South Africa [17]. More recent studies in the USA and UK among Family Medicine residents and general practitioners, respectively, reached the same result [8,18]. These findings are likely attributable to insufficient ECG training within Family Medicine curricula. This conclusion is also shared by a study, which emphasized the need to improve the nursing curriculum in terms of ECG training because of the low competency they achieved [10].

Numerous studies have reported similarly low ECG interpretation competency among medical students, nurses, paramedics, and other healthcare professionals [1,11,17,19,20,21,22]. This finding raises concern and has prompted national medical authorities to investigate the underlying causes and improve medical curricula accordingly. Because ECG interpretation is a vital skill, especially for frontline providers such as emergency medical service personnel, nurses, Family Medicine physicians, and general practitioners, these professionals must receive adequate training to achieve proficiency. Comparing different studies on ECG interpretation is challenging due to variations in both the electrocardiograms themselves and the methodologies used. This variability may account for the wide discrepancies in reported interpretation accuracy, which ranged from 17% to 63% in a systematic review by Salerno et al. covering papers published between 1996 and 2002 [23].

Interestingly, a study by Santos et al. included three general practitioners and showed a high competency, reaching 81% [22]. Although this is a positive result, the study does not acknowledge the very limited sample size it encompassed. Furthermore, it is mentioned that those general practitioners are involved in academic teaching of family medicine physicians, which is the likely reason for their high competency. Finally, the proposed ECGs were collected from patients’ clinical files in the primary care center, which means a lack of objective assessment of ECG interpretation, and that most of the ECGs are likely to be normal.

Another interesting finding is that 65.8% of the participants in the study couldn’t identify the normal ECG pattern. This finding is also shared by Amini et al., who showed that a normal ECG was the second least common pattern to identify [1]. This has a significant impact on the healthcare system in terms of cost and unnecessary referrals, and in terms of patients’ psychological stress from a wrong diagnosis. Again, this re-emphasizes the importance of providing adequate ECG training to physicians, focusing also on identifying normal patterns of ECGs.

In a statement on ECG competency, the American College of Cardiology (ACC) and the American Heart Association (AHA) emphasized the need for a systematic method to measure ECG interpretation skills [15]. They noted that no established approach exists for assessing and credentialing physicians in this area, aside from cardiology board certification, which applies only to cardiology residents. Consequently, they recommended that standardized ECG exams, such as the ABIM ICE ECG exam, serve as the benchmark for granting ECG interpretation privileges to qualified physicians.

Regarding participants’ perceptions of facilitators and barriers to ECG learning, the majority reported that practical, case-based training is a key facilitator. This finding is supported by a study comparing online case-based discussion with online lecture-based teaching, which demonstrated the superiority of the case-based discussion in enhancing ECG competency among trainee doctors [24]. Further research is needed to address this gap in the literature and identify strategies that can effectively improve ECG training in medical curricula.

Surprisingly, our study revealed that completion of the ACLS course was associated with improved ECG competency in univariate analysis. However, after adjusting for experience, professional level, and other educational exposures in the final logistic regression model, this association was reduced and no longer statistically significant, although the odds ratio remained above 1. This pattern suggests that ACLS may contribute to better ECG performance as part of a broader bundle of training opportunities rather than as an isolated factor. Our finding is broadly in line with studies reporting a beneficial role of ACLS training, even though one study did not demonstrate a significant impact [8,10,11,25]. This disparity could be due to the population of participants in these studies. The studies of Mobrad et al., Aljohani et al., and ours included paramedic students from the Emergency Medical Services College, critical care nurses, and Family Medicine residents with general practitioners, respectively [10,25]. Due to the nature of their specialty and the frequent exposure to ECGs, enrollment in the ACLS course conferred significant improvement in ECG competency.

Another notable finding in our study is that completing ECG courses and receiving teaching during clinical rotations were both associated with higher ECG competency in the univariate analysis, echoing the results of previous work [1,5,12,20,25,26]. This improvement is likely due to the focused learning that these educational activities provide. However, in the fully adjusted model, these variables did not remain independent predictors, which suggests that their effects may overlap with other forms of structured ECG teaching, particularly regular ECG lectures and specialist-level training. In contrast, attendance in ECG lectures exhibited a significant association both in univariate and multivariate analyses, proving its efficacy in the association with higher ECG competency, which is supported by previous studies [5,12].

Seniority showed a more heterogeneous pattern in our study. Participants with 5–10 years of experience were associated with significantly decreased ECG competency, compared with those with more than 10 years of experience. In contrast, those with less than 5 years did not differ significantly. In addition, family medicine specialists had a significantly higher probability of achieving good ECG competency than general practitioners, while consultants and residents did not differ significantly from general practitioners. These mixed findings partly echo studies that reported better performance among more senior participants [1,4,5,12]. Whereas others did not find a clear relationship between seniority and ECG competency [8,14,18,26]. Differences in study populations and in how seniority is defined may account for these inconsistencies.

The present study offers several strengths. First, it is the first to assess ECG competency among Family Medicine doctors at all levels and general practitioners in the Eastern Province of Saudi Arabia, with participants drawn from multiple primary healthcare centers, which confers a fair amount of generalizability. Second, it employed a validated and reliable questionnaire to evaluate ECG competency, lending greater credibility to the results. Third, it is one of the few studies examining the impact of ACLS courses on overall ECG competency, providing valuable insights for medical education authorities. Nonetheless, there are limitations to consider. First, the use of the convenience sampling method limits how far the reported prevalence can be generalized beyond this specific sample. Second, because physicians completed the survey independently, there is a possibility that they consulted external resources when answering. Finally, the cross-sectional design prevents establishing any causal relationships between variables.

The aforementioned results, especially the alarming increase in wrong identification of normal ECGs, mandate specific and targeted improvements to the educational element of physicians in healthcare systems. Consequently, this study brought several key insights and recommendations for medical education authorities to improve ECG interpretation competency. First, the ACLS course is a preferred element to be integrated into the training curriculum for Family Medicine residents. The best way to integrate it is by allocating a specific week or month dedicated to studying the course and making this certificate a requirement for Family Medicine Board certification. As for Family Medicine specialists and consultants, and general practitioners, the best way to integrate the ACLS course is by making its certification a prerequisite for practicing medicine. Second, ECG courses, teaching during clinical rotations, and, more importantly, ECG lectures are the most important facilitators reported by Family Medicine doctors for enhancing ECG skills. The best way to integrate it is by making it part of the rotations of the Family Medicine Board and as academic activities that are held every period in the healthcare facility, as part of continuous academic teaching for general practitioners. Finally, adding a standardized ECG examination to the medical license exam with recertifications would be an important addition, especially for physicians whose roles involve ECG interpretation and mandating. Although ECG courses, teaching during clinical rotation, ACLS course, and ECG standardized examination were not significant in the multivariate analysis or were not even analyzed, previous literature showed a positive effect for integrating them as facilitators for ECG competency.

## 5. Conclusions

Our study aimed to evaluate ECG interpretation competency among primary healthcare physicians and identify factors that facilitate or hinder learning. It demonstrated generally poor competency, with practical, case-based training cited as the most significant facilitator and, paradoxically, inadequate exposure to hands-on methods reported as the chief barrier. Participation in the ACLS course, ECG courses, and clinical rotation teaching activities was significantly associated with higher levels of competency. These findings underscore a pressing need to strengthen ECG interpretation skills among frontline physicians. Key recommendations include incorporating the ACLS course into their curriculum and regularly administering standardized ECG examinations. Future research should involve larger and more diverse populations, employ longitudinal designs to uncover potential causes of poor competency, and use robust sampling and data-collection methods to bolster reliability. Collectively, such efforts will expand our understanding of the challenges surrounding ECG interpretation and help develop evidence-based interventions that address these shortcomings.

## Figures and Tables

**Table 1:** Participant’s characteristics and main source of knowledge.

Item		*N*	*N*%
Gender	Male	117	45.5%
	Female	140	54.5%
Experience years	Less than 5 years	152	59.1%
	5–10 years	52	20.2%
	More than 10 years	53	20.6%
Level	Family medicine resident	101	39.3%
	Family medicine specialist	36	14.0%
	Family medicine consultant	43	16.7%
	General practitioner	77	30.0%
Education (program) sector	Al-Ahsa Health Cluster	102	39.7%
	Johns Hopkins Aramco Healthcare Center in Dhahran	24	9.3%
	Eastern Health Cluster	73	28.4%
	King Fahad University Hospital in Khobar	28	10.9%
	Health Services Program in the Royal Commission in Jubail	8	3.1%
	Other	22	8.6%
Cardiology rotation	Yes	133	51.8%
	No	124	48.2%
ACLS	Yes	127	49.4%
	No	130	50.6%

ACLS; Advanced Cardiovascular Life Support.

**Table 2:** Participant’s level of knowledge based on the questionnaire overall score.

Knowledge Level		
Classification	*N*	*N*%
Good (≥7.5)	118	45.9%
Poor (<7.5)	139	54.1%

**Table 3:** Participants ‘s perspective toward ECG learning facilitators and barriers (multiple options can be reported).

Facilitators	*N* (*N*%)	
	Yes	No
Practical case-based training in the program	177 (68.9%)	80 (31.1%)
Extracurricular educational courses	127 (49.4%)	130 (50.6%)
Volunteer work in cardiology clinics	70 (27.2%)	187 (72.8%)
Cardiology block important aspects should be adopted and implanted in all blocks	119 (46.3%)	138 (53.7%)
Other	39 (15.2%)	218 (84.8%)
**Barriers**	***N* (*N*%)**	
	**Yes**	**No**
Inadequate training in the program	177 (68.9%)	80 (31.1%)
Dependence on cardiologist expertise	86 (33.5%)	171 (66.5%)
ECG interpretation itself is difficult	120 (46.7%)	137 (53.3%)
Lack of resources	78 (30.4%)	179 (69.6%)
Other	10 (3.9%)	247 (96.1%)

**Table 4:** Crosstab association between participant’s characteristics and their source of knowledge and their level of knowledge.

		Good	Poor	*p*-Value
		*N* (%)		
Gender	Female	67 (47.9%)	73 (52.1%)	0.494
	Male	51 (43.6%)	66 (56.4%)	
Experience (years)	5–10 years	15 (28.8%)	37 (71.2%)	0.016 *
	Less than 5 years	74 (48.7%)	78 (51.3%)	
	More than 10 years	29 (54.7%)	24 (45.3%)	
Level	Family medicine consultant	21 (48.8%)	22 (51.2%)	0.274
	Family medicine resident	46 (45.5%)	55 (54.5%)	
	Family medicine specialist	21 (58.3%)	15 (41.7%)	
	General practitioner	30 (39.0%)	47 (61.0%)	
Cardiology rotation	Yes	65 (48.9%)	68 (51.1%)	0.324
	No	53 (42.7%)	71 (57.3%)	
ACLS	Yes	68 (53.5%)	59 (46.5%)	0.015 *
	No	50 (38.5%)	80 (61.5%)	
Source of Knowledge				
Attendance in regular ECG lectures	Yes	66 (53.2%)	58 (46.8%)	0.023 *
	No	52 (39.1%)	81 (60.9%)	
Teaching during Clinical Rotations	Yes	65 (52.4%)	59 (47.6%)	0.043 *
	No	53 (39.8%)	80 (60.2%)	
ECG courses	Yes	59 (53.2%)	52 (46.8%)	0.042 *
	No	59 (40.4%)	87 (59.6%)	
Self-study using printed materials (Textbooks)	Yes	45 (45.5%)	54 (54.5%)	0.907
	No	73 (46.2%)	85 (53.8%)	
Self-study using web-based sources (online)	Yes	66 (49.3%)	68 (50.7%)	0.262
	No	52 (42.3%)	71 (57.7%)	

* Statistically significant (*p* < 0.05). ACLS; Advanced Cardiovascular Life Support.

**Table 5:** Multivariable binary logistic regression analysis for Predictors of Good ECG Knowledge.

Predictor		B	*p*-Value	Odds Ratio Exp(B)	95% CI for Exp(B)
Experience	Less than 5 years	−0.226	0.521	0.798	0.400–1.589
	5–10 years	−1.054	0.014 *	0.349	0.151–0.806
	More than 10 years	Reference			
ACLS		0.627	0.022 *	1.872	1.093–3.207
Attendance in regular ECG lectures		0.498	0.070	1.646	0.960–2.822
Teaching during clinical rotations		0.436	0.106	1.547	0.911–2.626
ECG courses		0.407	0.128	1.503	0.890–2.538

Model Summary: χ^2^(6) = 24.611, *p* < 0.001; −2 Log Likelihood = 329.95; Nagelkerke R^2^ = 0.122. The Hosmer–Lemeshow test indicated good model fit (χ^2^ = 9.808, *p* = 0.279). Statically Significant Values *p*-Value (>0.05) marked with “*”.

## Data Availability

The raw data supporting the conclusions of this article will be made available by the authors on request.

## Supplementary Materials

The following supporting information can be downloaded at: https://www.mdpi.com/article/10.3390/healthcare13233040/s1. File S1: STROBE-Checklist, File S2: ECG-Questionnaire.

## Abbreviations

The following abbreviations are used in this manuscript:

ECG	Electrocardiogram
ACLS	Advanced Cardiac Life Support
CVD	Cardiovascular disease
STROBE	Strengthening the Reporting of Observational Studies in Epidemiology
