# Supplementary material for: Electrocardiogram Interpretation Competency of Primary Health Care Physicians: A Cross-Sectional Study
Source: Healthcare (Basel). 2025 Nov 25;13(23):3040. doi: 10.3390/healthcare13233040 (PMC12692582; doi:10.3390/healthcare13233040)
Supplement: Supplementary file 1 [file healthcare-13-03040-s001.zip › healthcare-3940019-supplementary.pdf]

Supplementary material (File S1): STROBE Statement—checklist of items that should be included in reports of observational studies

	Item No.	Recommendation	Page No.	Relevant text from manuscript
**Title and abstract**	1	(*a*) Indicate the study's design with a commonly used term in the title or the abstract	1	Title and abstract
		(*b*) Provide in the abstract an informative and balanced summary of what was done and what was found	1	Abstract
**Introduction**				
Background/rationale	2	Explain the scientific background and rationale for the investigation being reported	2	Introduction, Paragraph 1
Objectives	3	State specific objectives, including any prespecified hypotheses	2	Introduction, Paragraph 3
**Methods**				
Study design	4	Present key elements of study design early in the paper	2	Methods, Paragraph 1-2
Setting	5	Describe the setting, locations, and relevant dates, including periods of recruitment, exposure, follow-up, and data collection	2-3	Methods, Paragraph 1-2
Participants	6	(*a*) *Cohort study*—Give the eligibility criteria, and the sources and methods of selection of participants. Describe methods of follow-up<br>*Case-control study*—Give the eligibility criteria, and the sources and methods of case ascertainment and control selection. Give the rationale for the choice of cases and controls<br>*Cross-sectional study*—Give the eligibility criteria, and the sources and methods of selection of participants	2-3	Methods, Paragraph 2-3
		(*b*) *Cohort study*—For matched studies, give matching criteria and number of exposed and unexposed<br>*Case-control study*—For matched studies, give matching criteria and the number of controls per case		
Variables	7	Clearly define all outcomes, exposures, predictors, potential confounders, and effect modifiers. Give diagnostic criteria, if applicable	3	Methods, Paragraph 5-6
Data sources/ measurement	8*	For each variable of interest, give sources of data and details of methods of assessment (measurement). Describe comparability of assessment methods if there is more than one group	3	Methods, Paragraph 4
Bias	9	Describe any efforts to address potential sources of bias	2	Methods, Paragraph 2
Study size	10	Explain how the study size was arrived at	2-3	Methods, Paragraph 3

Continued on next page

Quantitative variables	11	Explain how quantitative variables were handled in the analyses. If applicable, describe which groupings were chosen and why	3	Methods, Paragraph 7
Statistical methods	12	(*a*) Describe all statistical methods, including those used to control for confounding		
		(*b*) Describe any methods used to examine subgroups and interactions		
		(*c*) Explain how missing data were addressed		
		(*d*) *Cohort study*—If applicable, explain how loss to follow-up was addressed<br>*Case-control study*—If applicable, explain how matching of cases and controls was addressed<br>*Cross-sectional study*—If applicable, describe analytical methods taking account of sampling strategy	3	Methods, Paragraph 7
		(*e*) Describe any sensitivity analyses		
**Results**				
Participants	13*	(a) Report numbers of individuals at each stage of study—eg numbers potentially eligible, examined for eligibility, confirmed eligible, included in the study, completing follow-up, and analysed	3-4	Results, Paragraph 1
		(b) Give reasons for non-participation at each stage		
		(c) Consider use of a flow diagram		
Descriptive data	14*	(a) Give characteristics of study participants (eg demographic, clinical, social) and information on exposures and potential confounders	4-5	Results, Figure 1, Paragraph 2-10, Table 1-4,
		(b) Indicate number of participants with missing data for each variable of interest		
		(c) *Cohort study*—Summarise follow-up time (eg, average and total amount)		
Outcome data	15*	*Cohort study*—Report numbers of outcome events or summary measures over time		
		Case-control study—Report numbers in each exposure category, or summary measures of exposure		
		Cross-sectional study—Report numbers of outcome events or summary measures	5	Results, Paragraph 7-8, Figure 2
Main results	16	(*a*) Give unadjusted estimates and, if applicable, confounder-adjusted estimates and their precision (eg, 95% confidence interval). Make clear which confounders were adjusted for and why they were included	9	Results, Paragraph 11, Table 5
		(*b*) Report category boundaries when continuous variables were categorized		
		(*c*) If relevant, consider translating estimates of relative risk into absolute risk for a meaningful time period		

Continued on next page

Other analyses	17	Report other analyses done—eg analyses of subgroups and interactions, and sensitivity analyses		
**Discussion**				
Key results	18	Summarise key results with reference to study objectives	10	Discussion, Paragraph 7
Limitations	19	Discuss limitations of the study, taking into account sources of potential bias or imprecision. Discuss both direction and magnitude of any potential bias	12	Discussion, Paragraph 13
Interpretation	20	Give a cautious overall interpretation of results considering objectives, limitations, multiplicity of analyses, results from similar studies, and other relevant evidence	12-13	Discussion, Paragraph 14
Generalisability	21	Discuss the generalisability (external validity) of the study results	12	Discussion Paragraph 4
**Other information**				
Funding	22	Give the source of funding and the role of the funders for the present study and, if applicable, for the original study on which the present article is based	12	Conclosion, Paragraph 4

*Give information separately for cases and controls in case-control studies and, if applicable, for exposed and unexposed groups in cohort and cross-sectional studies.

Note: An Explanation and Elaboration article discusses each checklist item and gives methodological background and published examples of transparent reporting. The STROBE checklist is best used in conjunction with this article (freely available on the Web sites of PLoS Medicine at http://www.plosmedicine.org/, Annals of Internal Medicine at http://www.annals.org/, and Epidemiology at http://www.epidem.com/). Information on the STROBE Initiative is available at www.strobe-statement.org.

Supplementary material (File S2):

Electrocardiogram interpretation competency of primary Health care physicians in the Eastern province in Saudi Arabia

Dear Primary Health care Physicians,

We are conducting this study to test the competence and confidence in ECG interpretation of Primary Health care Physicians. The results will aid in assessing the Primary Health care Physicians and faculty performance in the Eastern Province in Saudi Arabia. This questionnaire is **anonymous**. We highly emphasize on honesty in responses, and we truly appreciate your participation.

Section 1: Consent

Do you agree to participate in this research by answering this questionnaire?

- □ No
- □ Yes

Section 2: Demographic data

Gender:
Male.
Female.

Experience years:
Less than 5
5-10 years
More than 10

Level
Residents

Specialist
Consultants
General practitioners

Which of the following sources primarily contributed to your ECG interpretation skills: (you can choose more than one)*

1-Attendance in regular ECG lectures.
2- Teaching during Clinical Rotations.
3- Teaching during Clinical Rotations.
4-Teaching during Clinical Rotations.
5- ECG courses.
Self-study using printed materials (Textbooks). Self-study using web-based sources (online).

Have you previously/currently taken cardiology as part of your training rotations?
□ Yes
□ No

Did you finish ACLS course?
- □ No
- □ Yes

Did you do any training course about electrocardiography? (Do not consider EMS 312)
- □ No
- □ Yes (if affirmative, answer the following questions)
  - a. When was the last course?
    - i. < 1 year or 1 year
    - ii. Between 2-5 years
    - iii. More than 5 years
  - b. How was the course taken?
    - i. Online
    - ii. Face-to-face
    - iii. Hybrid

c. How many hours was the course?

i. < 10 hours

ii. 10-20 hours

iii. >20 hours

Section 4: ECG-based cases

1. What is the correct order of EKG waves and intervals?

A. P wave, QRS complex, T wave, PR interval, ST interval, U wave

B. T wave, P wave, QRS complex, PR interval, ST interval, U wave

C. QRS complex, P wave, PR interval, T wave, ST interval, U wave

D. I do not know

2. If in an EKG the p wave does not appear, what is your first thought?

A. There is a conduction problem between the ventricles

B. There is a conduction problem between the atriums

C. It is normal, it does not have to appear in an EKG

D. I do not know

3. You perform an EKG and observe this register. What do you think it might be?

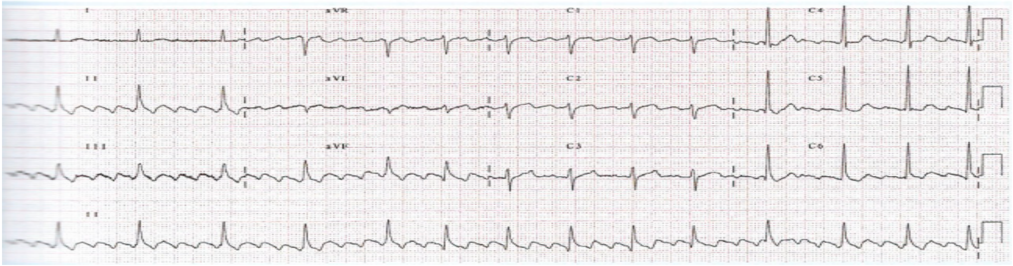

A. A third degree heart block
B. An atrial flutter
C. A supra-ventricular tachycardia
D. I do not know

4. You perform an EKG and observe this register. How would you act?

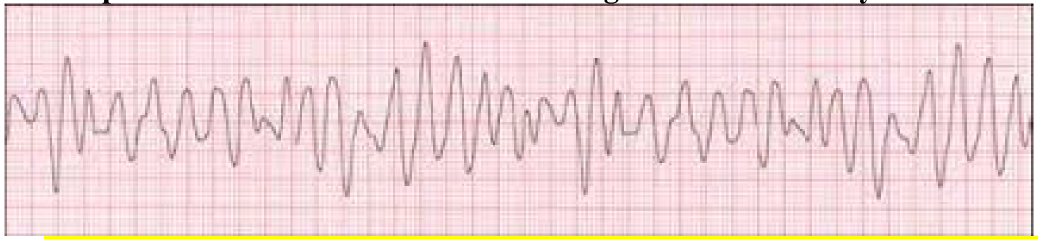

A. Ask for help without leaving the patient alone because it is a ventricular fibrillation
B. Ask for help without leaving the patient alone because it is an atrial fibrillation
C. Perform another EKG because it looks like there may be interference
D. You do not know how to act but you know it must be a serious problem

5. A patient comes to the Emergency Department due to a respiratory distress. He has 140 beats per minute. You perform an EKG and observe the following:

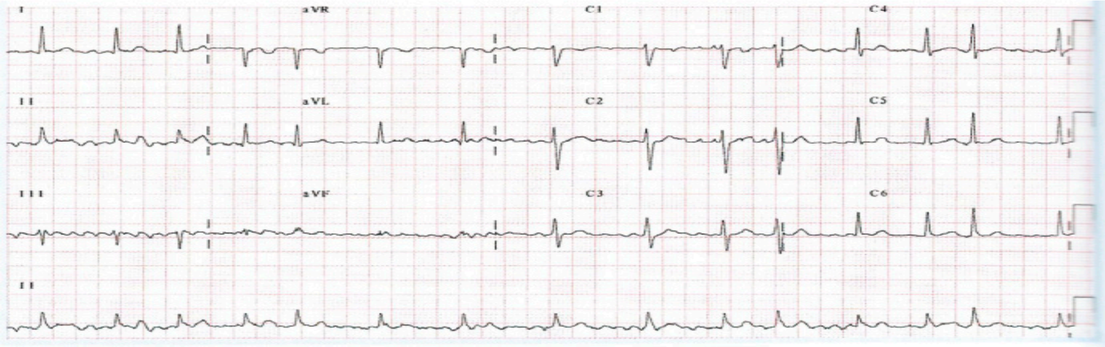

A. It is an atrial tachycardia
B. It is an atrial fibrillation
C. It is an atrial extra-systole
D. I do not know

6. A patient with precordial pain for more than 8 hours. You perform a 12-branch EKG. After observing the EKG, what catches your attention?

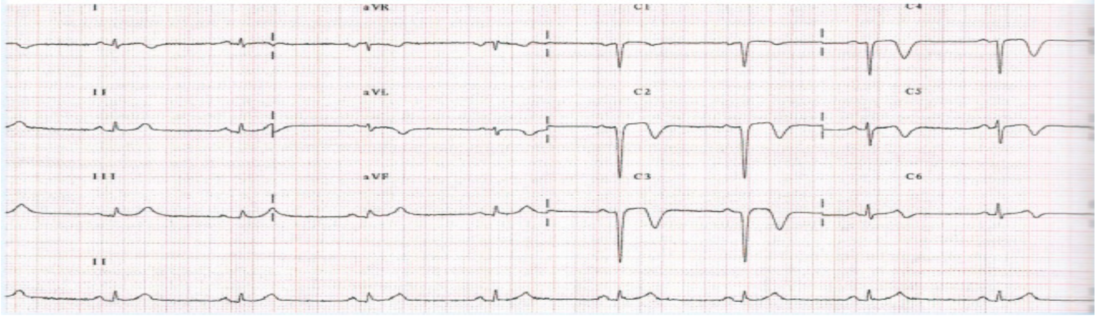

A. You can see pathological pauses

B. You can see pathological Q waves
C. The patient has a low cardiac rhythm
D. I do not know

7. What pathology you think the patient with this EKG has?

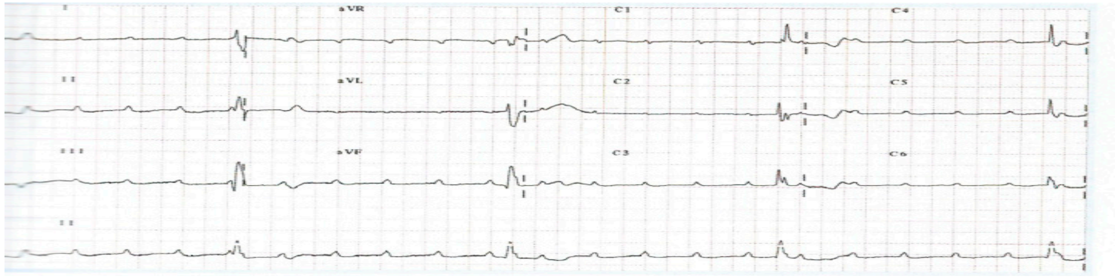

A. A first-degree heart block
B. He does not have any pathology
C. A third-degree heart block
D. I do not know.

8. A hospitalized patient who had had surgery due to an AMI is transferred to the Emergency Department to be monitored because his vital signs are unstable. You perform an EKG and observe the following:

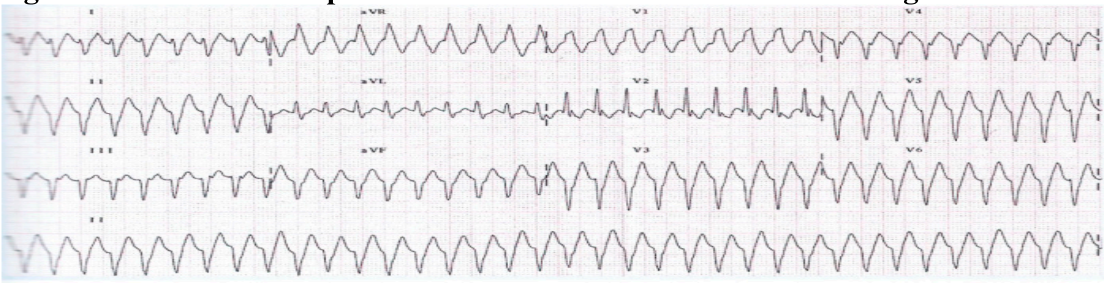

A. The patient presents a ventricular tachycardia
B. The patient presents a supra-ventricular tachycardia
C. The patient presents an atrial tachycardia
D. I do not know

9. You are in triage and call a patient who reports medium-intensity precordial pain. He tells you that the pain appeared after leaving an important meeting two hours ago. He is 52 years of age and hypertensive and a few months ago he was diagnosed with Diabetes Mellitus II. You perform a 12-branch EKG and observe the following:

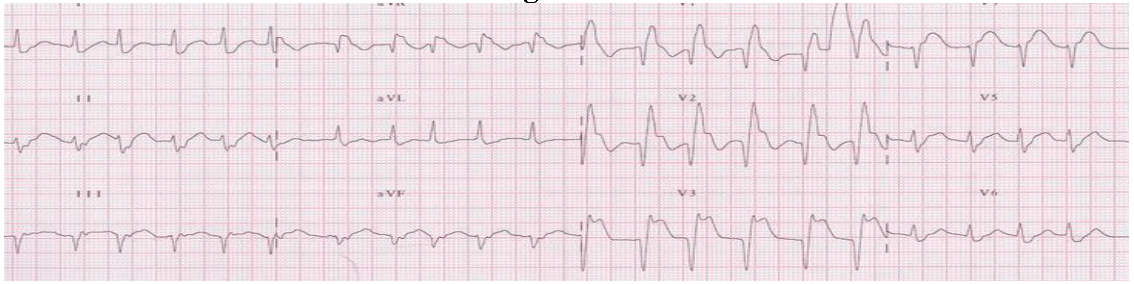

A. It is a supra-ventricular tachycardia
B. It is ST elevation myocardial infarction
C. It is an acute myocardial infarction with a pathological Q wave
D. I do not know.

10. A 24-year-old male comes to the Emergency Department He is athletic and slim. He reports feeling a pricking sensation in the left area of his chest since he finished doing exercise (3 hours earlier). You perform an EKG and observe the following:

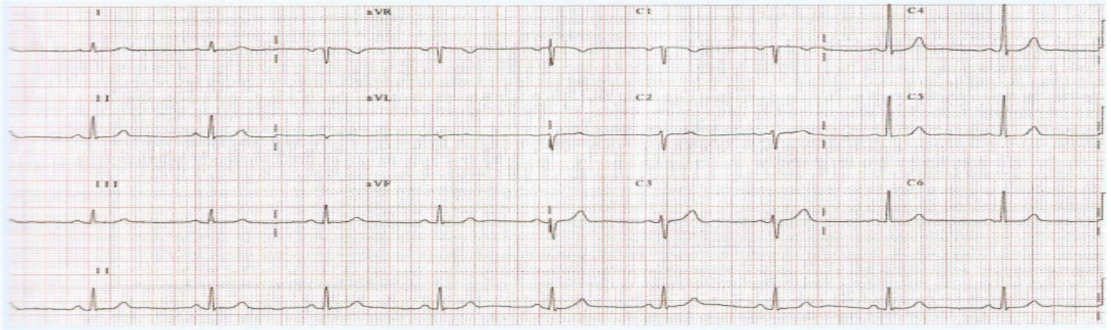

A. It is an atrial bradycardia
B. He has conduction problems
C. It is a normal EKG
D. I do not know

11. A patient with digitalis intoxication comes from a hospitalization ward. Before monitoring him you perform an EKG and obtain the following:

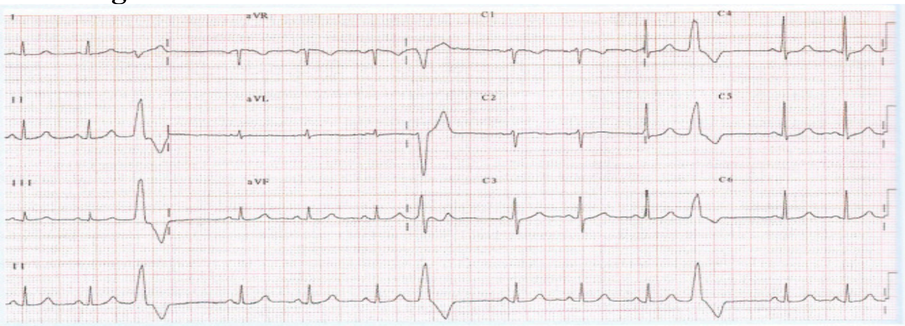

A. You observe an atrial extra-systole
B. You observe a ventricular extra-systole
C. You observe that he is carrying a pacemaker
D. I do not know

12. A 30-year-old woman comes to the Emergency Department reporting palpitations, chest tightness and dyspnea. You perform an EKG and observe the following:

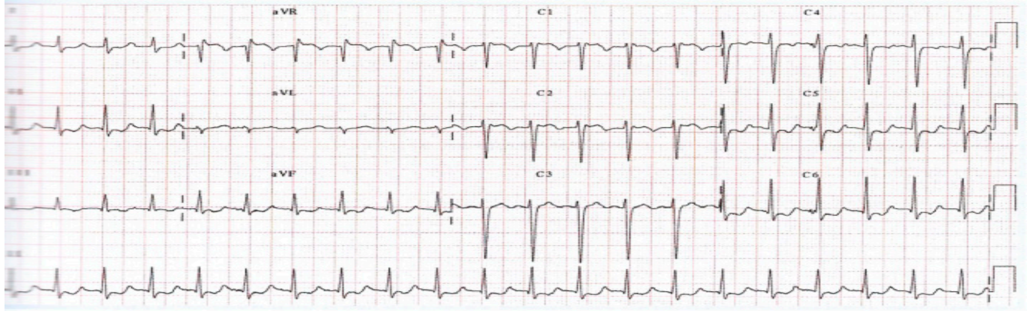

A. It is a ventricular tachycardia
B. It is an atrial extra-systole
C. It is an atrial tachycardia
D. I do

Section 5: Primary Health care physicians perspective

In your opinion, what could facilitate Saudi medical interns to develop competent ECG interpretation skills?

□ Practical case-based training in college
□ Extracurricular educational courses
□ Volunteer work in cardiology clinics
□ Cardiology block important aspects should be adopted and implanted in all blocks
□ Other

In your opinion, what are the limitations facing Saudi medical interns from developing competent ECG interpretation skills?

□ Inadequate training in college
□ Dependence on cardiologist expertise
□ ECG interpretation itself is difficult
□ Lack of resources
□ Other

Notes:

- **Correct answer to each question is highlighted in yellow**

Reference

- Coll-Badell M, Jiménez-Herrera MF, Llaurado-Serra M. Emergency nurse competence in electrocardiographic interpretation in Spain: a cross-sectional study. *J Emerg Nurs*. 2017;**43**:560–570. doi: 10.1016/j.jen.2017.06.001
